# A comparative wordlist for the languages of The Gran Chaco, South America

**DOI:** 10.12688/openreseurope.14922.2

**Published:** 2022-12-06

**Authors:** Nicolás Brid, Cristina Messineo, Johann-Mattis List

**Affiliations:** 1 Universidad de Buenos Aires, Buenos Aires, Argentina; 2 CONICET, Buenos Aires, Argentina; 3 Department of Linguistic and Cultural Evolution, Max Planck Institute for Evolutionary Anthropology, Leipzig, 04103, Germany

**Keywords:** South American languages – Gran Chaco – comparative wordlist – structural borrowing

## Abstract

Home to more than twenty indigenous languages belonging to six linguistic families, the Gran Chaco has raised the interest of many linguists from different backgrounds. While some have focused on finding deeper genetic relations between different language groups, others have looked into similarities from the perspective of areal linguistics. In order to contribute to further research of areal and genetic features among these languages, we have compiled a comparative wordlist consisting of translational equivalents for 326 concepts — representing basic and ethnobiological vocabulary — for 26 language varieties. Since the data were standardized in various ways, they can be analyzed both quantitatively and qualitatively. In order to illustrate this in detail, we have carried out an initial computer-assisted analysis of parts of the data by searching for shared lexicosemantic patterns resulting from structural rather than direct borrowings.

## (Plain language summary)

In this data note we present a list of words in indigenous languages of the Gran Chaco region in South America. These languages belong to arguably six established language families, whose deeper relationship is under discussion. Five of those language families are found only in the Gran Chaco, while one of them, the Tupi-Guarani family, is found across all of South America. In order to make it easy to compare the words in the wordlist, we standardized the data in several ways. We illustrate how the data can be analyzed by providing examples for cases in which words in unrelated languages show similar structures without being directly borrowed from each other.

## Introduction

The Gran Chaco is a South American eco-region that extends through north-central Argentina, eastern Bolivia, western Paraguay and southern Brazil. It is located north of the Salado river, east of the Andes mountains, south of the Amazon, from which it is separated by the Chiquitania, and west of the Paraguay and Paraná rivers. Apart from languages that have entered the region through conquest and colonization, such as Spanish, German and Paraguayan Guaraní, the region is home to indigenous languages of six different families: Guaicuruan, which includes Toba, Western Toba, Pilagá, Mocoví, Kadiwéu and extinct Abipón; Matacoan or Mataguayan, which includes Wichí, Maká, Nivaclé, and Chorote; Enlhet-Enenlhet, which includes Enlhet, Enxet, Enenlhet, Guaná, Sanapaná and Angaité; Zamucoan, which includes Ayoreo and Chamacoco; Lule-Vilela, which includes only Lule and Vilela; and Tupi-Guarani, which in the Gran Chaco includes Tapiete, Ava, and Guaraní Izoceño but which also extends all through South America ( Campbell & Grondona, 2012; Durante, 2018; Fabre, 2005; Golluscio & Vidal, 2010). For many of these languages there are also different geographic varieties.

The linguistic diversity of the Gran Chaco and the striking similarities in the features of some apparently unrelated languages have attracted the attention of numerous linguists, who have approached the topic from various theoretical and methodological frameworks. On the one hand, much research has focused on genetic relations among the languages. Recently, for instance, it has been stated that Vilela and extinct Lule are related and the family has been named Lule-Vilela ( Viegas Barros, 2001), or that Guaicuruan and Matacoan languages have a common genetic origin and belong to one family, termed Guaicuruan-Matacoan ( Viegas Barros, 1993; Viegas Barros, 2013a). Previous work had proposed even greater language family groupings ( Kaufman, 1990; Mason, 1950). On the other hand, similarities among Chaco languages, not only Guaicuruan and Matacoan, have been analysed from the perspective of areal linguistics. Such similarities include phonological traits such as the presence and absence of certain phonemes, as well as grammatical features like the presence of possessive classifiers and noun determiners ( Comrie *et al*., 2010).

Fewer studies, however, have focused on shared semantic features that are visible in the lexicon in the form of similar lexical motivation patterns ( Campbell & Grondona, 2012; Messineo *et al*., 2010). In that sense, we consider that a big-scale dataset for further comparison of the Gran Chaco languages is a necessary tool that we have been lacking. Even though there have been many valuable works that compare different languages of the region, some of the criteria are inconsistent, and they seldom deal with the entirety of the indigenous languages of the Gran Chaco in a human and machine-readable way. Such an enterprise should be a starting point for a project that includes genetic comparison and concrete investigation of both lexical and pattern borrowing across Chaco languages of different families.

## Materials and methods

### Materials

**Figure 1.**
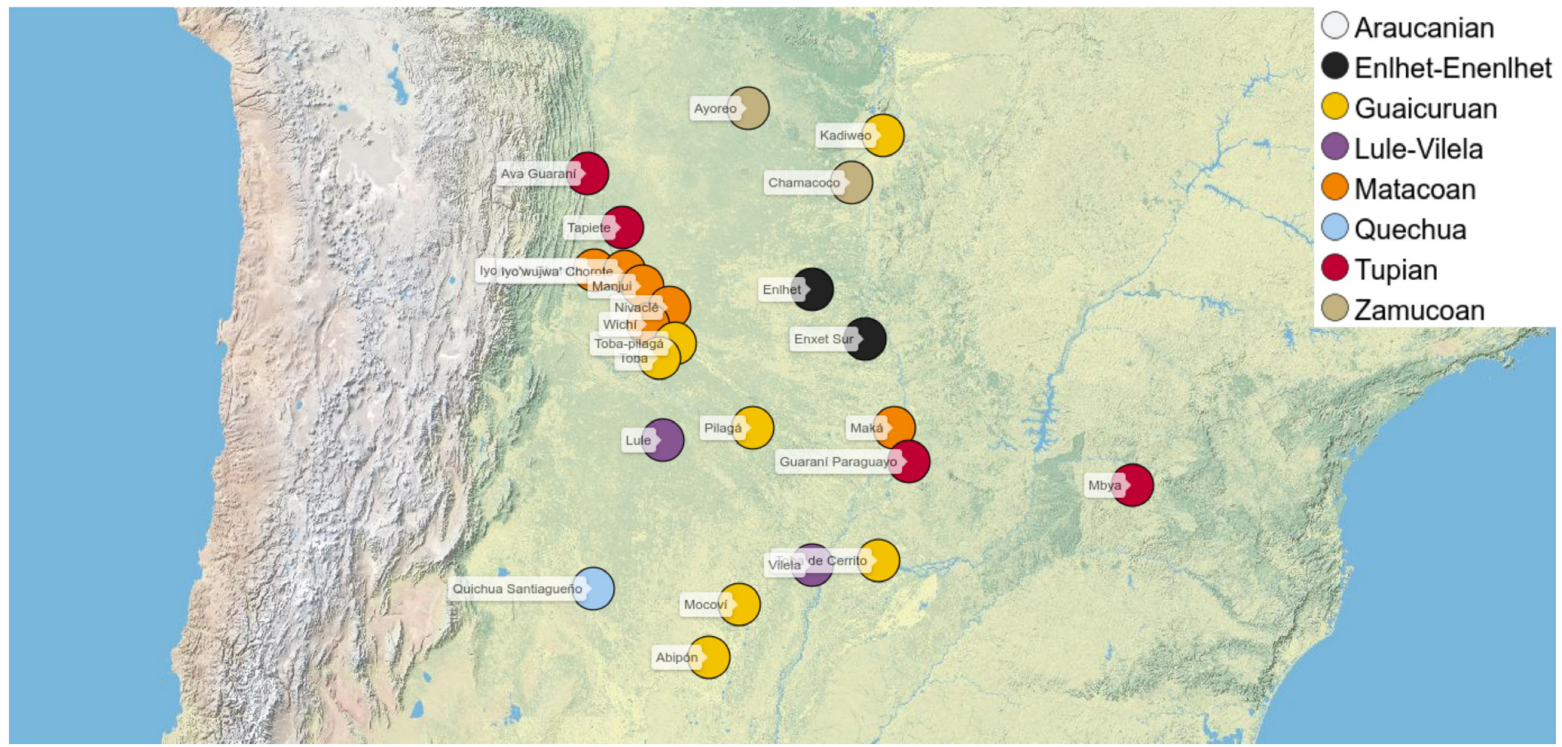
Languages covered in our study (with exception of Mapudungun, which is located further in the South).

Two different datasets were first individually compiled and later combined for this study. The first one comprised a list of 502 concepts reflecting basic vocabulary terms translated into 23 language varieties spoken in the Chaco area and two language varieties from other regions. The second one consisted of 825 ethnobiological concepts translated into 16 Chaco varieties. While the coverage for the basic dataset was rather high, with most languages showing word forms for 80% and more of the data, the coverage for the ethnobiological dataset was rather low, since the terms are highly specific and it was often difficult to find translations for all terms in resources available for the respective varieties. In order to allow for a more targeted comparison of the languages with respect to lexical structures, we then decided to combine them. This decision was motivated by the fact that — although previous research showing interesting cases of pattern borrowing in flora and fauna vocabulary had sparked our interest in that domain — we realized that the lexical motivation for the formation of individual terms still depends to a large degree on words and morphemes that can primarily be found in the realm ofbasic vocabulary. Thus, a combined list, albeit imperfect, permits a detailed study on pattern borrowing while taking lexical motivation patterns into account. For this purpose, we selected 224 concepts from the basic vocabulary lists, and 100 ethnobiological concepts, resulting in a total of 324 concepts for 23 language varieties (see Table 1), which are geographically distributed across and around the Chaco area (see Figure 1).

**Table 1.** Languages and data points covered in our study.

#	Variety	Family	F	C	B	E	Co	Sources
1	Abipón	Guaicuruan	216	155	155	0	0.48	Najlis, 1966
2	Ava Guaraní	Tupian	263	215	215	0	0.66	Dietrich, 2021 (IDS)
3	Ayoreo	Zamucoan	377	228	212	16	0.70	Benz & Salinas Jacai Picanerai, 2020; Briggs, 2021 (IDS); Schmeda-Hirschmann, 1998
4	Chamacoco	Zamucoan	251	163	162	1	0.50	Ulrich & Ulrich (2000)
5	Enlhet	Enlhet-Enenlhet	438	252	216	36	0.78	Arenas, 1981; Unruh & Kalisch, 1997
6	Enxet Sur	Enlhet-Enenlhet	334	209	189	20	0.65	Rojas & Curtis, 2017
7	Guaraní Paraguayo	Tupian	325	238	214	24	0.73	Carol, 2018; Guasch & Ortiz, 1986; Seelwische, 1980
8	Iyojwa'ja Chorote	Matacoan	360	274	216	58	0.85	Drayson, 2009; Scarpa, 2010
9	Iyoʼwujwa Chorote	Matacoan	254	190	176	14	0.59	Carol, 2018
10	Kadiweo	Guaicuruan	225	158	157	1	0.49	Griffiths, 2002; Sándalo, 1995
11	Lule	Lule-Vilela	296	174	174	0	0.54	Machoni & Larsen, 1877
12	Maká	Matacoan	282	243	199	44	0.75	Arenas, 1983; Gerzenstein, 1999
13	Mapudungun	Araucanian	256	207	207	0	0.64	Fernández Garay *et al.,* 2021
14	Mbya	Tupian	223	168	168	0	0.52	Cadogan, 1992
15	Mocoví	Guaicuruan	298	216	213	3	0.67	Buckwalter & Ruiz, 2021; Rosso, 2010
16	Nivaclé	Matacoan	376	250	217	33	0.77	Seelwische, 1980
17	Pilagá	Guaicuruan	287	248	211	37	0.77	Buckwalter & Suárez, 2021; Filipov, 1993; Vidal, 2010 and Vidal, 2013
18	Quichua Santiagueño	Quechua	235	176	162	14	0.54	Bravo, 1975
19	Tapiete	Tupian	272	202	194	8	0.62	González, 2005; González, 2011
20	Toba	Guaicuruan	471	273	216	57	0.84	Buckwalter & Litwiller de Buckwalter, 1980; Buckwalter & Sánchez, 2021; Cúneo & Porta, 2009; Martínez, 2009
21	Toba de Cerrito	Guaicuruan	180	154	154	0	0.48	Messineo, 2009
22	Toba-pilagá	Guaicuruan	368	255	192	63	0.79	Arenas, 1993; Tebboth, 1943
23	Wichí	Matacoan	388	241	209	32	0.74	Braunstein, 2021 (IDS), DIWICA (2021); Suárez, 2010 and Suárez, 2014

Column F refers to the forms in the data, column C refers to the concepts that are covered, columns B and E refer to the number of concepts covered from basic and ethnobiological vocabulary, and column Co refers to the coverage (number of attested concepts divided by number of concepts in the whole wordlist).

The collection of basic words was compiled from various sources, mainly dictionaries, but in some cases also from grammatical descriptions. One of the largest contributors was the Intercontinental Dictionary Series (IDS), ( Key & Comrie, 2021). Other material came from individual sources available for the respective varieties, mainly dictionaries, wordlists, and compilations of different Chaco languages. In these cases, translational equivalents for the basic words were carried out manually. The collection of ethnobiological terms was typically compiled from specific lists of ethnobiological vocabulary, taken from articles and books dedicated to the topic, but in some cases, unified resources for basic vocabulary and ethnobiological terms were available and could be used.

### Methods

In creating our resource, we had two major goals in mind. On the one hand, we wanted to create a resource that is both human- and machine-readable at the same time, allowing us to analyse the data and annotate particular findings step by step in future work (this process is ongoing work and might be featured in studies to be published in the future). On the other hand, we wanted to create a resource that can be easily compared with other lexical resources, both on a world-wide and a regional scale. This allows us to make use of additional information or to compare our findings with those reported for other areas of the world in our future work. In order to achieve the first goal, we used an internal representation of the data for analysis and annotation, based on the Etymological Dictionary Edictor ( EDICTOR, Version 2.0, List, 2021a), in which we curate the data manually, annotating the data for various aspects, such as cognacy, borrowings, or borrowed patterns (loan translations) shared across the Chaco languages. In order to achieve the second goal, we converted our data to Cross-Linguistic Data Formats ( CLDF, Forkel *et al*., 2018), using the Lexibank workflow for the curation of lexical data in CLDF ( List *et al*., 2022a). While data curation and annotation with the help of the EDICTOR tool were largely done in a manual fashion, the conversion to CLDF was mostly done automatically, providing additional steps that helped us to identify potential problems in our data.

### Data curation with EDICTOR

Basic vocabularies and ethnobiological vocabularies were first collected separately. Only later, when we realized that both can be better analyzed in combination, we decided to combine them. For this purpose, we decided for a combined list of 324 items, with 224 basic vocabulary items and 100 ethnobiological items in total. Both datasets were combined to form a single TSV file in the format required by the EDICTOR tool and converted to an SQLITE database, using the PyEdictor package ( List, 2021b, Version 0.4), which we use to allow for the convenient online editing of the data.

Our main intention for the analysis was to annotate structural borrowings, that is, cases of borrowings in which it is not the word form that is being transferred, but rather the lexical motivation by which certain objects can be denoted. As an example, consider the English term “(computer) mouse”, which is reflected as *ratón de computadora* (literally “mouse or rat of the computer”) in Spanish.

In order to annotate structural borrowings in the Chaco data, we made use of existing annotation schemes that were developed for the handling of partial cognates ( Hill & List, 2017) and later extended to handle more complex cases of language-internal cognates and semantic shift ( Schweikhard & List, 2020) and ultimately implemented in Version 2.0 of the EDICTOR tool ( List, 2021a). The main idea of these annotation schemes is to provide what we call ‘morpheme glosses’ for each word form in the data and combine these with identifiers for partial cognates (see List *et al*., 2016).

As an example, consider the words for “beak” and “lip” for Maká and Chorote (both from the Matacaon language family) and Pilaga (from the Guaicuruan family) in Table 2. As can be seen from the table, all three language varieties express the word for “beak” by using the entire word or a part of the word for “lip”. Since Pilaga is not related with Chorote and Maká, and the form that expresses the concept “lip” in Pilaga ([a s e p], according to our annotation) is not cognate with the form [p a s] in Chorote and Maká, we assign these forms different cognate set identifiers (2 for [a s e p] and 4 for [p a s]). But since we judge the pattern as identical, consisting of a possessive marker (marked as :poss in our morpheme glosses) and the reuse of the form “lip” to denote the concept “beak”, we assign them the same pattern identifier, indicating that we have a shared structure here. Whether this structural commonality is due to language contact or due to independent processes of lexical change cannot be said at this point, since the pattern annotation is work in progress and has not been done for all of the data. Assembling more of these patterns in our data, however, will eventually allow us to find out whether these scenarios might result from contact or not.

**Table 2.** Example of our extended annotation of cognate sets, with morpheme glosses and structural similarities with respect to the motivation structure of individual word forms.

Family	Language	Concept	Form	Cognates	Structure	Morpheme Glosses
Guaicuruan	Pilaga	beak	n - a s e p	1 2	1 2	:poss **lip**
Guaicuruan	Pilaga	lip	n - a s e p	1 2	1 2	:poss **lip**
Matacoan	Chorote	beak	x i - p a s - a t	3 4 5	1 2 3	:poss **lip** :suff
Matacoan	Chorote	lip	x i - p a s - a t	3 4 5	1 2 3	:poss **lip** :suff
Matacoan	Maká	beak	ɬ a - p a s	6 4	1 2	:poss **lip**
Matacoan	Maká	lip	p a s	4	2	l **ip**

Table 2 shows words for “beak” and “lip” across three varieties from two language families. While word forms are not cognate across the two language families, and also not borrowed directly, we find structural similarities with respect to the motivation. In all three varieties, our annotation assumes that the word for “beak” is derived from the word for “lip”. We indicate this structural commonality with the help of identifiers that reflect the abstract structure (column Structure) and with the help of morpheme glosses, that provide an analysis of the underlying motivation (column Morpheme Glosses). Note that our analysis is not the only possible one for the given data. One could likewise argue or speculate that the word for “beak” was primary and that the word for “lip” was derived from it. In this case, the morpheme glosses would have to be modified. In order to avoid being forced to make a decision on the primary word form, one can — finally — also use neutral morpheme glosses like “beak/lip” which would explicitly avoid to make any judgment regarding primary or secondary word forms in the data.

### Data Sharing with CLDF

Whenever substantial changes to the data have accumulated and we decide to release a new version, we export the dataset and convert it automatically to CLDF. In doing so, we carry out several consistency checks of the data and make sure that the individual datapoints are maximally comparable across datasets from different sources. The CLDF conversion is carried out with the help of the CLDFBench toolkit that offers a command line interface that facilitates the conversion of language data to CLDF formats ( Forkel & List, 2020, https://pypi.org/project/cldfbench). Since we are working with lexical data, we additionally use the PyLexibank plugin for CLDFBench (Forkel *et al*., 2021), which offers extended functionality (see List *et al*., 2022a). The conversion to CLDF makes sure that our concepts are regularly linked to the most recent version of the Concepticon reference catalogue ( List *et al*., 2022b), that all languages, where possible, are linked to Glottolog ( Hammarström *et al*., 2022), and that the transcriptions follow the standards proposed by the Cross-Linguistic Transcription Systems reference catalogue ( List *et al*., 2021). Since the CLDF standard currently does not (yet) offer standards to annotate structural borrowings, we define custom formats for now (see Table 2), which we will propose for the inclusion in future versions of CLDF. In the following, we discuss the integration of our data with the three reference catalogs of (Concepticon, Glottolog, and CLTS) in more detail. 

***Concept linking.*** The concept list underlying our study was linked to the Concepticon reference catalogue (Version 2.6, List *et al*., 2022b). Concepticon offers unique identifiers for various concepts that are frequently used in questionnaires for language documentation and historical language comparison. Since Concepticon is by now more and more often used as a common standard reference for lexical datasets, also underlying large collections such as the Database of Cross-Linguistic Colexifications (CLICS) ( Rzymski *et al*., 2020) or the Lexibank repository of standardized wordlists in CLDF formats ( List *et al*., 2022a), we also made sure to link the concepts in our data to Concepticon, where possible. For the very specific plant and animal names in our data, however, the Concepticon does not offer concept identifiers. Here, we therefore linked our data to the Global Biodiversity Information Facility (GBIF). 

***Language mapping.*** Another way of linking the data with already existing sources consists in the linking of language varieties to the Glottolog project ( Hammarström *et al*., 2022). Glottolog provides unique identifiers for several language varieties, including dialect points and ancient varieties along with additional information regarding the language families to which the respective languages belong. For two varieties in our data, no Glottocode could be found. These are Manjui, which is a variety of Chorote spoken in the territory of Paraguay, and Toba de Cerrito, also spoken in the Paraguayan Chaco. These have not been identified as separate varieties on Glottolog yet, but might be added in future versions.

Most of the languages in our dataset are spoken in the Gran Chaco region of South America, in the territories of Argentina, Bolivia, Brazil, and Paraguay. In addition, we have chosen three languages spoken in adjacent regions, which we hope to use as control cases in future analyzes, namely Mapudungun (Araucanian), spoken in southern Chile and Argentina, Mbyá (Tupí-Guaraní), spoken in Argentina, Brazil and Paraguay, and Quichua Santiagueño (Quechuan), spoken in north-central Argentina. Although we are aware that these languages are spoken in the vicinity of the Gran Chaco, their inclusion as control languages responds to the fact that we intend to find shared semantic patterns that are not even found in adjacent territories. However, while some patterns have been observed in our data only in the Gran Chaco languages, others do appear also in the control languages. While it is true that areal influence does not end abruptly, and thus those coincidences could also be due to language contact exceeding the limits of the Gran Chaco, this could also be explained by the fact that not all shared semantic patterns are equally ubiquitous, with some patterns being more likely shared due to common typological traits in the world’s languages. This point, and the need for a hierarchy on pattern borrowing in order to rank the evidence by strength, is discussed in the conclusion. Even so, future studies should include control languages spoken in additional locations (in and out of South America) in order to render the results more robust. Finally, Paraguayan Guaraní is usually not considered a Chaco language in origin, but it has an undeniable influence on indigenous communities of the Gran Chaco, especially in the territory of Paraguay, where it is the second and sometimes the first language of many indigenous people who are multilingual in other languages.

When searching for the translational equivalents of individual concepts in our concept lists in the different sources for the varieties we included in our sample, it is often difficult to decide which word corresponds best to a given concept, specifically in cases where one has to choose from several variants. Variants may result from several reasons. On the one hand, two translations for the same concept may correspond to different varieties that have been included in the same resource. For example, we have added a document for a variety of Toba spoken in Paraguay, Toba de Cerrito. However, this variety has two subvarieties, one spoken in the village of Rioverde and the other spoken in the village of Rosario. In those cases in which these subvarieties display different forms, we indicate in a comment which form corresponds to which variety. In future versions of the database, we plan to find more principled ways of handling this kind of dialectal variation. On the other hand, different resources may give different forms for the same concept but no indication in which regard the forms differ (e.g., regarding their usage, specific semantic nuances, etc.). In these cases we indicated the different sources in our comments, but hope to find a more principled way to handle these cases of variation in future versions of our database.

This study includes Lule and Abipón, two extinct varieties of which no speakers are known to have survived until today. The original sources of these varieties were written by missionaries in the eighteenth and nineteenth centuries. Since transcription practices differed largely in the past, we cannot fully account for the accuracy of the transcriptions we used. Including the varieties in the study has proven useful, however, since it allowed us to check whether certain kinds of semantic patterns existed already 300 or 200 years before.

***Phonetic transcriptions.*** After having compiled the vocabulary in the corresponding sheets, the forms were converted, into a broad version of the International Phonetic Alphabet, called B(road)IPA, the central transcription system underlying the five transcription systems provided in the CLTS reference catalog. For the initial conversion, we made use of orthography profiles ( Moran & Cysouw, 2018), which are integrated into the Lexibank workflow for the curation of lexical data, which we used for our study ( List *et al*., 2022a). In this workflow, original forms are preserved, and for the target phonetic transcriptions used for cross-linguistic comparison, automatic tests are carried out to make sure they only reflect sounds defined in the CLTS reference catalog.

The conversion of transcription systems used by individual scholars to standardized transcriptions that conform to CLTS can be considerably tedious, especially when different transcription systems are underlying the data from every source. The conversion therefore required an intensive study of the phonological descriptions of all language varieties in our sample, for which often information often could only be found in broader grammatical descriptions. Inspecting the data also revealed that our initial conversion to phonetic transcriptions with orthography profiles was at times not optimal or contained occasional errors, which we then had to refine manually by modifying the data in the EDICTOR application. For the two extinct languages in our collection, Lule and Abipón, no reliable phonological descriptions available. In the case of Abipón, we followed the description of on phonology in Viegas Barros (2013b), based on comparison with other Guaicuruan languages. For Lule, we followed Zamponi’s analysis from 2008.

### Implementation

**Figure 2.**
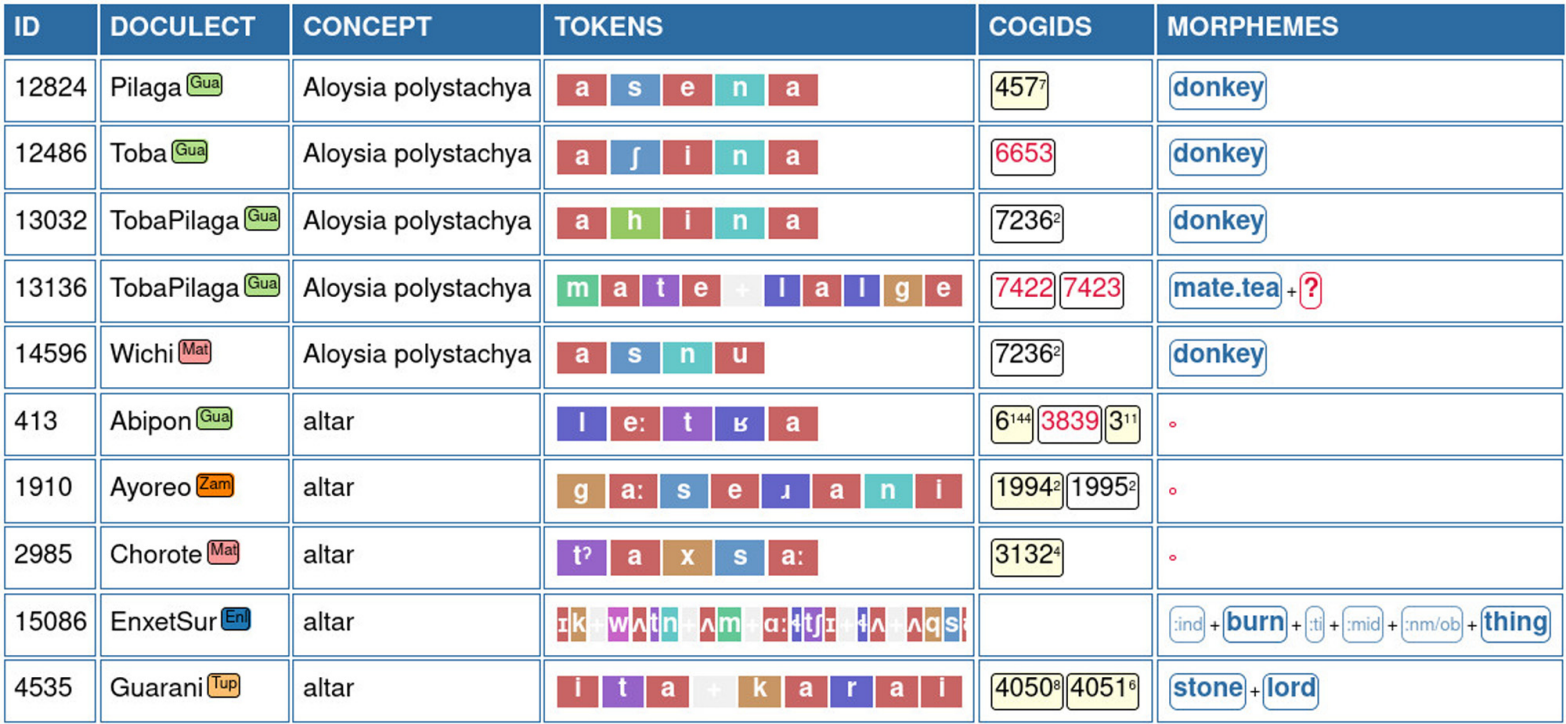
Curating the data with the help of the EDICTOR interface. The screenshot shows the Wordlist panel view of the EDICTOR tool. Word forms are rendered by coloring speech sounds according to their major sound class.

Having set up the data in its current form, our workflow for data curation and analysis now consists of two steps. In a first step, the data is analyzed using the EDICTOR tool. Figure 2 shows how the data appear in the Wordlist panel of the EDICTOR interface. In order to share the data publicly, we then used the Lexibank workflow ( List *et al*., 2022a) to convert the data automatically into Cross-Linguistic Data Formats, which can be triggered from the commandline. The conversion automatically checks various aspects of the data, including the transcriptions as reflected in a given version of the CLTS reference catalog, the mapping to a given Glottolog version and a given Concepticon version, and the formal correctness of currently available annotations.

## Conclusion

Although we consider the collection of the dataset reported here as preliminary, it has reached a stage where we can start with the concrete analysis of individual patterns in the data ( Brid *et al*., 2022). In the future, we plan to enhance the current dataset further and also extend the annotation of cognate words and structural borrowings.

Although we consider the dataset as good enough to publish it at this point, we should make clear that we are not fully content with all decisions we undertook in the past when collecting our data. By explicitly pointing to these points of dissatisfaction, we hope that we can warn readers of this study to avoid our mistakes when conducting similar works.

Firstly, we warn future researchers against mixing multiple sources for the same language varieties with no overt indication. For instance, our Chorote, Wichí, and Ayoreo data come from different sources. Although it may be important to include multiple sources, it would be advantageous to include a reference to the source in the database, perhaps in a separate column. This would make a discussion of the data and the underlying decisions which led to their creation more transparent. Also, it may turn out that a source differs from another source because it is based on a different language variety, perhaps more in contact with another language of the region. In suchhh a case, having that information at one’s disposal would be highly relevant for the results.

Even if sources are overtly indicated, a future reader would have to find the entries in the source. However, at present our data is not visible in its original orthography. For that reason, we encourage similar projects in the future to keep the original transcription in a separate column. This would enable users to copy-paste the original form in order to look it up in the original source. We plan to solve these two issues in the future, but at this stage, our data curation process had advanced too much to allow us for handling these problems efficiently.

Finally, it would also be desirable to rank the evidence for borrowingby strength. This means that, in order to address the topic of areal influence on shared semantic patterns, one would like to be able to tell the difference between patterns that may be shared due to typological traits common to the world’s languages and patterns that are more likely shared due to areal influence. This requires a theoretical and methodological apparatus that permits to suppose some kind of hierarchy on pattern borrowing. Since — to the best of our knowledge — such an apparatus does not exist at the moment, we can only hope on future research to provide us with additional tools to enhance the analysis of our datasets.

## Ethics and consent statement

Ethical approval and consent were not required.

## Data Availability

Data and Software available from: https://github.com/lexibank/chacolanguages/ Archived source code and data at time of publication: https://doi.org/10.5281/zenodo.6660368 License: Creative Commons Attribution 4.0 International license (CC-BY 4.0)

## References

[ref-1] Arenas P : Etnobotánica lengua-maskoy [Lengua-Maskoy ethnobotanics]. Buenos Aires: Fundación para la Educación, la Ciencia y la Cultura.1981.

[ref-2] Arenas P : Nombres y usos de las plantas por los indígenas Maká del Chaco Boreal [Names and uses of plants by the Maká Indians of the Chaco Boreal].In: *Parodiana.*Buenos Aires: Asociación Parodiana.1983;2(2):131–229. Reference Source

[ref-3] Arenas P : Fitonimia toba-pilagá [Toba-Pilagá phytonymy].In: Braunstein, José and Messineo, Cristina (eds.), *Hacia una nueva carta étnica del Gran Chaco V.*Las Lomitas, Formosa: Centro del Hombre Antiguo Chaqueño.1993;75–100.

[ref-5] Benz EA Salinas Jacai Picanerai J : Diccionario Ayoeode Uuode – Español – Español – Ayoeode Uuode [Ayoreo – Spanish dictionary]. Asunción: Fondo Nac6ional de la Cultura y las Artes.

[ref-7] Braunstein J : Wichí dictionary. In: Key Mary Ritchie and Comrie, Bernard (eds.) *The Intercontinental Dictionary Series.*Leipzig: Max Planck Institute for Evolutionary Anthropology.2021. Reference Source

[ref-8] Bravo D : Diccionario quichua santiagueño-castellano [Santiago del Estero Quichua – Spanish dictionary]. Buenos Aires: Editorial Universitaria de Buenos Aires.1975. Reference Source

[ref-21] Brid N List JM Messineo C : Las lenguas del Chaco desde la perspectiva de la semántica léxica. Análisis preliminar de patrones léxicos compartidos en el dominio etnobiológico [The languages of the Gran Chaco from the perspective of lexical semantics. Preliminary analysis of shared lexical structures in the ethnobotanical domain].. *LIAMES.* 2022;22: e022005,1–21. 10.20396/liames.v22i00.8669038

[ref-9] Briggs J : Ayoreo dictionary. In: Key Mary Ritchie and Comrie, Bernard (eds.) *The Intercontinental Dictionary Series.*Leipzig: Max Planck Institute for Evolutionary Anthropology.2021. Reference Source

[ref-10] Buckwalter A Litwiller de Buckwalter L : Vocabulario toba.Buenos Aires: Talleres Gráficos Grancharoff.1980. Reference Source

[ref-11] Buckwalter A Sánchez O : Toba dictionary. In: Key Mary Ritchie and Comrie, Bernard (eds.) *The Intercontinental Dictionary Series.*Leipzig: Max Planck Institute for Evolutionary Anthropology.2021. Reference Source

[ref-12] buckwalter A Ruiz R : Mocoví dictionary. In: Key Mary Ritchie and Comrie, Bernard (eds.) *The Intercontinental Dictionary Series.*Leipzig: Max Planck Institute for Evolutionary Anthropology.2021. Reference Source

[ref-13] Buckwalter A Suárez J : Pilagá dictionary. In: Key Mary Ritchie and Comrie, Bernard (eds.) *The Intercontinental Dictionary Series.*Leipzig: Max Planck Institute for Evolutionary Anthropology.2021. Reference Source

[ref-14] Cadogan L : Diccionario mbyá guaraní – castellano [Mbya Guarani - Spanish dictionary]. Asunción: CEADUC.1992. Reference Source

[ref-15] Campbell L Grondona V : Languages of the Chaco and Southern Cone.In: *The indigenous languages of South America: A comprehensive guide.*Berlin: De Gruyter Mouton.2012;2:625–667. 10.1515/9783110258035.625

[ref-16] Carol J : Inamtes jleeizi' Inkijwas ji'lij - Kiláyi ji'lij: Diccionario Bilingüe Manjui - Castellano [Manjui –Spanish bilingual dictionary]. Asunción: Paraguái Ñe'ẽnguéra Sãmbyhyha.2018.

[ref-17] Comrie B Golluscio L Vidal A : El Chaco como área lingüística [Chaco as a linguistic area].In: *Estudios de lenguas amerindias.*Hermosillo, Sonora: Editorial Unison.2010;2:85–130. Reference Source

[ref-18] Cúneo P Porta A : Vocabulario toba sobre peces y aves [Toba vocabulary of fish and birds].In: Braunstein, José and Messineo, Cristina (eds.), *Hacia una nueva carta étnica del Gran Chaco.*Las Lomitas, Formosa: Centro del Hombre Antiguo Chaqueño.2009;VIII:237–252.

[ref-19] Dietrich W : Chiriguano dictionary. In: Key Mary Ritchie and Comrie, Bernard (eds.) *The Intercontinental Dictionary Series.*Leipzig: Max Planck Institute for Evolutionary Anthropology.2021. Reference Source

[ref-20] DIWICA: Wichi-siwele lhayhilh / Diccionario wichí-castellano [Wichí –Spanish dictionary]. Formosa: INILSyT.2021. Reference Source

[ref-22] Drayson N : 'Niwak Samtis: Diccionario Iyojwa'ja 'Lij-Kilay 'Lij (Chorote-Castellano) [Chorote - Spanish dictionary]. In: Braunstein, José and Messineo, Cristina (eds.), *Hacia una nueva carta étnica del Gran Chaco*. Las Lomitas, Formosa: Centro del Hombre Antiguo Chaqueño.2009;VIII:91–174.

[ref-24] Durante S : La lengua ayoreo (familia zamuco), de la sintaxis al discurso: Documentación y descripción de una lengua amenazada [The Ayoreo language, from syntax to discourse: documentation and description of an endangered language]. Buenos Aires: Facultad de Filosofía y Letras.2018. Reference Source

[ref-25] Fabre A : Los pueblos del Gran Chaco y sus lenguas, primera parte: Los enlhet-enenlhet del Chaco Paraguayo [The Gran Chaco peoples and their languages, first part: the Enlhet-Enenlhet of the Paraguayan Chaco]. In: *Centro de Estudios Antropologicos. Suplemento Antropologico*. Asunción: Universidad Católica Nuestra Señora de la Asunción.2005;40(1):503–569. Reference Source

[ref-26] Fernández Garay A Catrileo M Ritchie Key M : Mapudungun dictionary. In: Key, Mary Ritchie and Comrie, Bernard (eds.) *The Intercontinental Dictionary Series*. Leipzig: Max Planck Institute for Evolutionary Anthropology.2021. Reference Source

[ref-28] Filipov A : Fitonimia pilagá [Pilaga phytonymy]. In: Braunstein, José and Messineo, Cristina (eds.), *Hacia una nueva carta étnica del Gran Chaco*. Las Lomitas, Formosa: Centro del Hombre Antiguo Chaqueño.1993;V:101–119.

[ref-27] Forkel R Greenhill SJ Bibiko HJ : PyLexibank. The Python Curation Library for Lexibank [Software, Version 2.8.2]. Leipzig: Max Planck Institute for Evolutionary Anthropology.2021. Reference Source

[ref-29] Forkel R List JM : CLDFBench. Give your Cross-Linguistic data a lift. In: N. Calzolari, F. Béchet, P. Blanche, K. Choukri, C. Cieri, T. Declerck, et al. (Eds.) *Proceedings of the Twelfth International Conference on Language Resources and Evaluation (LREC 2020)*. Paris: European Language Resources Association (ELRA).2020;6997–7004. Reference Source

[ref-30] Forkel R List JM Greenhill S : Cross-Linguistic Data Formats, advancing data sharing and re-use in comparative linguistics. *Sci Data.* 2018;5:180205. 10.1038/sdata.2018.205 30325347 PMC6190742

[ref-31] Gerzenstein A : Diccionario etnolingüístico maká-español [Ethnolinguistic Maká - Spanish dictionary]. Buenos Aires: Archivo de Lenguas Indoamericanas.1999. Reference Source

[ref-32] Golluscio L Vidal A : Recorrido sobre las lenguas del Chaco y los aportes a la investigación lingüística [The Chaco languages and their contribution to linguistic research]. In: *Amerindia*. Paris: Association d'Ethnolinguistique Amérindienne.2010;33/34:3–40. Reference Source

[ref-33] González H : A grammar of Tapiete (Tupi-Guarani). Doctoral dissertation, University of Pittsburgh.2005. Reference Source

[ref-34] González HA : Léxico etnobotánico tapiete (tupí-guaraní), lengua del Chaco argentino [Ethnobotanic vocabulary of Tapiete, a language of the Argentine Chaco]. *Indiana.* 2011;28:255–288. 10.18441/ind.v28i0.255-288

[ref-35] Griffiths G : Dicionário da língua Kadiwéu: Kadiwéu- Português, Português- Kadiwéu [Kadiwéu language dictionary]. Cuiabá: Sociedade Internacional de Linguística.2002. Reference Source

[ref-36] Guasch A Ortiz D : Diccionario Guaraní-Castellano Castellano-Guaraní [Guarani - Spanish dictionary]. Asunción: CEPAG.1986. Reference Source

[ref-81] Hammarström H Forkel R Haspelmath M : Glottolog 4.6. Leipzig: Max Planck Institute for Evolutionary Anthropology.2022. 10.5281/zenodo.6578297

[ref-37] Hill N List JM : Challenges of annotation and analysis in computer-assisted language comparison: A case study on Burmish languages. *Yearbook of the Poznań Linguistic Meeting.* 2017;3(1):47–76. 10.1515/yplm-2017-0003

[ref-38] Kaufman T : Language history in South America: What we know and how to know more. In: Payne, D. (Ed.). *Amazonian Linguistics: Studies in Lowland South American Languages*. Austin: University of Texas Press.1990;13–67. Reference Source

[ref-39] Key MR Comrie B : The Intercontinental Dictionary Series. Leipzig: Max Planck Institute for Evolutionary Anthropology.2021. Reference Source

[ref-82] List JM Anderson C Tresoldi T : Cross-Linguistic Transcription Systems. Version 2.1.0. Max Planck Institute for the Science of Human History: Jena.2021. 10.5281/zenodo.4705149

[ref-42] List JM Forkel R Greenhill S : Lexibank, A public repository of standardized wordlists with computed phonological and lexical features. *Sci Data.* 2022a;9(316):1–31. 10.1038/s41597-022-01432-0 35013360

[ref-40] List JM Lopez P Bapteste E : Using sequence similarity networks to identify partial cognates in multilingual wordlists. In: *Proceedings of the 54th Annual Meeting of the Association for Computational Linguistics (Volume 2: Short Papers)*, Berlin: Association of Computational Linguistics.2016;2:599–605. 10.18653/v1/P16-2097

[ref-41] List JM : EDICTOR. A web-based interactive tool for creating and editing etymological datasets.[Software, Version 2.0]. Leipzig: Max Planck Institute for Evolutionary Anthropology.2021a. Reference Source

[ref-80] List JM : PyEDICTOR. A tool for the quick manipulation of CLDF datasets. Leipzig: Max Planck Institute for Evolutionary Anthropology.2021b. Reference Source

[ref-43] List JM Tjuka A Rzymski C : CLLD Concepticon [Dataset, Version 2.6.0]. Leipzig: Max Planck Institute for Evolutionary Anthropology.2022b. 10.5281/zenodo.6560398

[ref-86] Machoni A Larsen JM : Arte y vocabulario de la lengua lule y tonocoté: compuestos con facultad de sus superiores. Buenos Aires: PE Coni.1877. Reference Source

[ref-45] Martínez G : Fitonimia de los tobas bermejeños (Chaco Central, Argentina) [Phytonymy of the Bermejo Tobas of the Argentine Central Chaco]. In: Braunstein, José and Messineo, Cristina (eds.), *Hacia una nueva carta étnica del Gran Chaco*. Las Lomitas, Formosa: Centro del Hombre Antiguo Chaqueño.2009;VIII:194–212. Reference Source

[ref-46] Mason JA : The Languages of South American Indians. In: *Handbook of South American Indians*. Washington: United States Government Printing Office.1950;6:189–215. Reference Source

[ref-47] Messineo C : Vocabulario toba de Cerrito (Paraguay) [Toba vocabulary of Cerrito, Paraguay]. In: Braunstein, José and Messineo, Cristina (eds.), *Hacia una nueva carta étnica del Gran Chaco*. Las Lomitas, Formosa: Centro del Hombre Antiguo Chaqueño.2009;VIII:253–269.

[ref-48] Messineo C Scarpa G Tola F : Léxico y categorización etnobiológica en grupos indígenas del Gran Chaco [Ethnobiological vocabulary and categorization among indigenous groups of the Gran Chaco]. Santa Rosa: Universidad Nacional de La Pampa.2010. Reference Source

[ref-49] Moran S Cysouw M : The Unicode Cookbook for Linguists: Managing writing systems using orthography profiles. Berlin: Language Science Press.2018. Reference Source

[ref-84] Najlis EL : Lengua abipona. Archivo de lenguas precolombinas Buenos Aires 1.1-2.1966. Reference Source

[ref-50] Rojas A Curtis T : Diccionario Enxet Sur [Enxet Sur dictionary]. Río Verde: Equipo de Traducción de Enxet Sur.2017.

[ref-51] Rosso C : Compilación y análisis preliminar de la fitonimia de la flora leñosa de comunidades mocovíes del sudoeste chaqueño [Compilation and preliminary analysis of woody flora phytonymy in Mocovi communities of Southwestern Chaco].In: Messineo, C., Scarpa, G y Tola, F. (comps.), *Léxico y categorización etnobiológica en grupos indígenas del Gran Chaco.* Santa Rosa: Universidad Nacional de La Pampa.2010;251–272.

[ref-52] Rzymski C Tresoldi T Greenhill S : The Database of Cross-Linguistic Colexifications, reproducible analysis of cross- linguistic polysemies.In: *Sci Data.* 2020;7(13):13. 10.1038/s41597-019-0341-x 31932593 PMC6957499

[ref-53] Sandalo MF : A Grammar of Kadiweu.Doctoral dissertation, University of Pittsburgh.1995. Reference Source

[ref-54] Scarpa G : Hacia una etnotaxonomía vegetal chorote II: Clasificación de las plantas entre las parcialidades iyojwa’ja y iyowujwa del Chaco argentino [Towards a Chorote vegetal ethnotaxonomy II: plant classification among the Iyojwa’ja and Iyowujwa groups of the Argentine Chaco].In: Messineo, C., Scarpa, G y Tola, F. (comps.), *Léxico y categorización etnobiológica en grupos indígenas del Gran Chaco.* Santa Rosa: Universidad Nacional de La Pampa.2010;157–198. Reference Source

[ref-55] Schmeda Hirschmann G : Etnobotánica Ayoreo. Contribución al estudio de la flora y vegetación del Chaco. XI. [Ayoreo ethnobotanics. Contribution to the study of the Chaco flora and vegetation. XI]. *Candollea.* 1998;53(1):1–50. Reference Source

[ref-56] Schweikhard NE List JM : Developing an annotation framework for word formation processes in comparative linguistics.In: *SKASE Journal of Theoretical Linguistics.* 2020;17(1):2–26. Reference Source

[ref-57] Seelwische J : Diccionario Nivaclé-Castellano [Nivacle – Spanish dictionary].Asunción: CEADUC.1980. Reference Source

[ref-58] Suárez ME : Fitonimia wichí de especies arbóreas y arbustivas del Chaco Semiárido salteño [Wichí phytonymy of trees and bushes of the semi-arid Chaco Salteño].In: Messineo, C., Scarpa, G y Tola, F. (comps.), *Léxico y categorización etnobiológica en grupos indígenas del Gran Chaco.* Santa Rosa: Universidad Nacional de La Pampa.2010;199–224. Reference Source

[ref-59] Suárez ME : Etnobotánica wichí del bosque xerófito en el Chaco Semiárido salteño [Wichi ethnobotanics of the xerophyte woods of the semi-arid Chaco Salteño].Don Torcuato: Autores de Argentina.2014. Reference Source

[ref-60] Tebboth T : Diccionario toba [Toba dictionary].In: *Revista del Instituto de Antropología de Tucumán.* Tucumán: Universidad Nacional de Tucumán.1943;3(2):33–221. Reference Source

[ref-61] Ulrich M Ulrich R : Diccionario Ɨshɨro (Chamacoco) – Español / Español – Ɨshɨro (Chamacoco) [Spanish – Chamacoco dictionary].Asunción: New Tribes Mission.2000.

[ref-62] Unruh E Kalisch H : Moya’ansaeclha’nengelpayvaam nengeltomha enlhet.Comunidad Enhlet.1997. Reference Source

[ref-64] Vidal A : Diccionario Trilingüe Pilagá-Español-Inglés Interactivo [Interactive trilingual dictionary Pilaga – Spanish – English].Formosa: EDUNAF.2010.

[ref-65] Vidal A : Enseñanza de la lengua pilagá [Pilaga language teaching].Formosa: EDUNAF.2013. Reference Source

[ref-66] Viegas Barros P : ¿Existe una relación genética entre las lenguas mataguayas y guaycurúes? [Is there a genetic relationship between Mataguayan and Guaicuruan languages?].In: Braunstein, José and Cristina Messineo (eds.), *Hacia una nueva carta étnica del Gran Chaco V.* Las Lomitas, Formosa: Centro del Hombre Antiguo Chaqueño.1993;193–213. Reference Source

[ref-67] Viegas Barros P : Evidencias de la relación genética lule-vilela [Evidence for the genetic relationship between Lule and Vilela]. *LIAMES: Línguas Indígenas Americanas.* Campinas: UNICAMP.2001;1(1):107–126. Reference Source

[ref-68] Viegas Barros P : La hipótesis de parentesco Guaicurú-Mataguayo: estado actual de la cuestión [The Mataguayo-Guaicuruan relatedness hypothesis: current state of affairs]. *Revista brasileira de linguística antropológica.* Brasilia: Universidade de Brasília.2013a;5(2):293–333. 10.26512/rbla.v5i2.16269

[ref-69] Viegas Barros P : Proto-Guaicurú: Una reconstrucción fonológica, léxica y morfológica [Proto-Guaicuruan: a phonological, lexical, and morphological reconstruction].Munich: Lincom Europa.2013b. Reference Source

[ref-70] Zamponi R : Sulla fonologia e la rappresentazione ortografica del lule. In: *Introducción de Riccardo Badini y Raoul Zamponi a Maccioni Antonio (2008 [1732]) Arte y Vocabulario de la Lengua Lule y Tonocoté, edición al cuidado de Riccardo Badini, Tiziana Deonette, Stefania Pineider, XXI-LVIII.* Cagliari: Centro di Studi Filologici Sardi.2008. Reference Source

