# Supplementary material for: A comparative wordlist for the languages of The Gran Chaco, South America
Source: Open Res Eur. 2022 Dec 6;2:90. Originally published 2022 Jul 21. [Version 2] doi: 10.12688/openreseurope.14922.2 (PMC10446078; doi:10.12688/openreseurope.14922.2)
Supplement: Peer review history

# Peer review: Reviewer response for version 2

**DOI:** 10.21956/openreseurope.16643.r30464

**Published:** 2023-01-18

**Authors:** Rik van Gijn

**Affiliations:** 1 Leiden University Centre for Linguistics, Leiden, The Netherlands

I am happy to change the status of the paper to Approved

Are sufficient details of methods and materials provided to allow replication by others?

Partly

Is the rationale for creating the dataset(s) clearly described?

Yes

Are the datasets clearly presented in a useable and accessible format?

Partly

Are the protocols appropriate and is the work technically sound?

Yes

Reviewer Expertise:

NA

I confirm that I have read this submission and believe that I have an appropriate level of expertise to confirm that it is of an acceptable scientific standard.

# Peer review: Reviewer response for version 1

**DOI:** 10.21956/openreseurope.16126.r29747

**Published:** 2022-10-10

**Authors:** Rik van Gijn

**Affiliations:** 1 Leiden University Centre for Linguistics, Leiden, The Netherlands

I think this is an interesting initiative, which fills a gap in the comparative linguistic research domain: assessing the similarities between semantic structure of lexemes across languages. I do have a few remarks, questions, and things that were not quite clear to me for consideration. 

 1. The word list

 The word list consists of basic vocabulary (the largest part) and another part which is ethnobotanical vocabulary. The paper mentioned that the authors realized that these two parts were best combined into one. Can you say more about the reason behind this, why is it better to combine them? And did or do you have any expectations wrt borrowability of semantic structure of basic vocabulary versus more peripheral (ethnobotanical) vocabulary. When it comes to form borrowing, the received wisdom is that basic vocabulary is more resistant to borrowing than flora and fauna vocabulary, but is there an equivalent expectation for structural borrowing?

 2. Semantic coding

 I understand that, in the example given, the words for lip and beak are both assigned to the concept LIP. The important pattern here seems to be that words for beak and lip are connected to one and the same concept, and it wasn't entirely clear to me what happens if two languages have one and the same underlying concept for both lip and beak, but in slightly different ways. I'll sketch two hypothetical scenarios. One (admittedly unlikely) scenario is that there might be independent evidence that in fact BEAK is the original meaning and that it is more truthful to connect both the words for lip and beak to the concept BEAK. Would that count as a full mismatch with the languages that connect both words to the concept of LIP? Another scenario would be that the word for lip is in fact connected to the concept MOUTH, e,g, the word for lip might semantically be something like OUTER MOUTH, this in turn may be extended to the word for beak, but in this case both words are connected to a third concept MOUTH. Is that also a full mismatch with a language that extends LIP to the word for beak?

 3. Types of semantic structural isomorphisms

 Related to the previous point, do I understand correctly that the obligatory presence of a possessive prefix with words for lip and beak in the example count as much for a match on structural borrowing as the fact that both words are connected to the concept LIP? It seems to me that one match is more indicative about past contact than the other 9in general: the more specific and unusual, the more informative). Or do you have ways to differentiate between different types of structural matches? 

 4. You mention a number of control languages. These are all spoken in the immediate vicinity of the Gran Chaco, and I don't think it can be excluded that there were contacts between speakers of the control languages and the target languages. So I wonder to what extent are these control languages. Are they meant to show a diminished number of commonalities, or are they meant to give a baseline of accidental commonalities? If the latter, I think the control language are not the best choices. In any case, it is good to make this clear.

 5. I wonder if it is not a little too early to publish this paper. The concluding remarks suggest that the coding scheme can still change considerably. I cannot tell how much it may change and to what extent it would make the present publication obsolete, so I leave this for the consideration of the authors.

Are sufficient details of methods and materials provided to allow replication by others?

Partly

Is the rationale for creating the dataset(s) clearly described?

Yes

Are the datasets clearly presented in a useable and accessible format?

Partly

Are the protocols appropriate and is the work technically sound?

Yes

Reviewer Expertise:

My research interests include South American languages, reconstructing the social history in South America, language typology. I feel confident to assess the conceptual set up of the paper, but not to assess the technical details of the implementation.

I confirm that I have read this submission and believe that I have an appropriate level of expertise to confirm that it is of an acceptable scientific standard, however I have significant reservations, as outlined above.

# Response

**Published:** 2022-10-12

**Authors:** Johann-Mattis List

**Affiliations:** University of Passau, Germany

We are very grateful for this thorough review and will try to address all points raised soon, hoping that our answers will be convincing and that our modified version of the article will properly address all points raised.

# Response

**Published:** 2022-12-02

**Authors:** Johann-Mattis List

**Affiliations:** University of Passau, Germany

We would like to express our deep gratitude to the reviewer for taking the time to check our study and providing very profound and important comments. We have tried to directly react to the major criticisms brought up by the reviewer in our revised version of the study. We list these in the following in the form of bullet points in which we partially quote the points brought up by the reviewer.

- "Can you say more about the reason behind this, why is it better to combine them? And did or do you have any expectations wrt borrowability of semantic structure of basic vocabulary versus more peripheral (ethnobotanical) vocabulary. When it comes to form borrowing, the received wisdom is that basic vocabulary is more resistant to borrowing than flora and fauna vocabulary, but is there an equivalent expectation for structural borrowing?"

While we were initially mostly interested in an investigation of Flora and Fauna vocabulary, since previous research indicated that the most interesting patterns of pattern borrowing could be found in this area of the lexicon, we later realized that the motivation of individual terms still depends to a large degree on words and morphemes that can primarily be found in the basic vocabulary. As a result, our combination of the two concept lists may not be perfect, but it provides an initial idea, how detailed studies on pattern borrowing that take lexical motivation patterns into account, can be carried out. We have tried to clarify this in the introduction to the Materials section of the study, where we added more information on the advantages of the combined collection of basic vocabulary along with ethnobotanical terms.

- "One (admittedly unlikely) scenario is that there might be independent evidence that in fact BEAK is the original meaning and that it is more truthful to connect both the words for lip and beak to the concept BEAK.  Would that count as a full mismatch with the languages that connect both words to the concept of LIP? Another scenario would be that the word for lip is in fact connected to the concept MOUTH, e,g, the word for lip might semantically be something like OUTER MOUTH, this in turn may be extended to the word for beak, but in this case both words are connected to a third concept MOUTH. Is that also a full mismatch with a language that extends LIP to the word for beak?"

This is a very good remark which emphasizes the importance of taking historical pathways of semantic change into account when trying to match patterns of lexical motivation. We agree that one could definitely argue that the underlying patterns have different origins, while our current annotation practice points to a very specific direction of change. In order to avoid this, however, we can also employ an annotation of the partial colexification patterns that does not make any decisions regarding the direction of semantic change and lexical motivation processes. In such an annotation, we would leave it open, which form (BEAK or LIP) we take as the primary one, and we would indicate this by using a gloss BEAK/LIP in both cases. While this is a very simple solution to account for the problems raised here, it is clear that it may not be satisfying. However, we assume that the reviewer will agree with us that it is in any case difficult to judge which direction of change would be more probable. We have added a statement in our example that emphasizes that there are different solutions than the ones we propose and which also points to the “neutral” solution of morpheme glossing as an alternative.

- "Related to the previous point, do I understand correctly that the obligatory presence of a possessive prefix with words for lip and beak in the example count as much for a match on structural borrowing as the fact that both words are connected to the concept LIP? It seems to me that one match is more indicative about past contact than the other (in general: the more specific and unusual, the more informative). Or do you have ways to differentiate between different types of structural matches?"

This is a very good point which we have not really thought through so far. It is clear that in theory, one should be able to rank the evidence. In this way, one could distinguish more surprising types of structural matches from less surprising ones and use this to indicate which one we consider as more likely than the others. However, at this stage in our analysis, where we are still trying to figure out the most transparent ways to analyze the data, we are not able to provide[4] We have, however, added a short paragraph in the final outlook of our study, where we indicate that it would be desirable to a) rank the evidence by strength, and to b) come up with some kind of a hierarchy on pattern borrowing that could guide the ranking process. any solutions for the ranking or for a systematic comparison of different types of commonalities and their respective force to provide strict evidence for pattern borrowing. [4] We have, however, added a short paragraph in the final outlook of our study, where we indicate that it would be desirable to a) rank the evidence by strength, and to b) come up with some kind of a hierarchy on pattern borrowing that could guide the ranking process.

- "You mention a number of control languages. These are all spoken in the immediate vicinity of the Gran Chaco, and I don't think it can be excluded that there were contacts between speakers of the control languages and the target languages. So I wonder to what extent are these control languages. Are they meant to show a diminished number of commonalities, or are they meant to give a baseline of accidental commonalities? If the latter, I think the control language are not the best choices. In any case, it is good to make this clear."

We agree that the control languages are not a good choice to serve as a baseline for chance commonalities. Instead, the hope was to show that the closeness of the languages in the Chaco area leads to more commonalities between Chaco languages than with languages which are still spoken in South America but not in direct contact (thus corresponding to the first scenario mentioned). Adding control languages that might serve to illustrate accidental commonalities is an idea that we should discuss in the future. [5] For now, we have tried to clarify that the control languages in the current study were included as examples of South American languages that are not spoken in the Chaco, in order to see to what degree the possibility of a Sprachbund in the Chaco area might have eased the large amount of pattern borrowings that can be found there.

# Peer review: Reviewer response for version 1

**DOI:** 10.21956/openreseurope.16126.r29839

**Published:** 2022-09-20

**Authors:** Joshua Birchall

**Affiliations:** 1 Department of Linguistics, University of New Mexico, Albuquerque, NM, USA

This data note describes a preliminary dataset of basic vocabulary and ethnobiological terms compiled for 23 languages from the Gran Chaco region of South America and neighboring areas. Few large-scale cross-linguistic lexical datasets are currently available on South American indigenous languages, and many primary sources can be difficult to access, making this work especially relevant and important. This rationale is clearly conveyed in the article. 

 In general, sufficient detail is provided in the text and in the references cited to replicate the workflow. However, one issue that is worth mentioning is that some forms are attributed to multiple sources, e.g. the entries for Ayoreo and Chorote. When combined with the absence of page numbers for where particular forms are located in their respective sources, and an absence of the original orthographic transcription of the form in its source, identifying the provenance of certain forms is somewhat more difficult than need be. The presence of the original transcription of the forms in the source material would further make the phonological retranscription procedure carried out by the authors more transparent and replicable. It may be advantageous to consider each record in the dataset as a particular instance of a documented form attributed to a particular semantic concept in a particular source, a 'docunym'. While this may produce multiple forms attributed to the same concept for a single language, adding further complexity to computational work, this would address some of the issues discussed by the authors regarding having "to decide which word corresponds best to a given concept" across different sources and different language varieties.

 The dataset as presented is useable and accessible to the target user, either as a reference for lexical information on the languages of the Gran Chaco or as a starting point for comparative analyses. Furthermore, the protocols adopted for the creation of this dataset make use of a suite of workflows, tools and reference catalogs that are not only appropriate, but help to define a standard for comparative lexical work within the field.

Are sufficient details of methods and materials provided to allow replication by others?

Partly

Is the rationale for creating the dataset(s) clearly described?

Yes

Are the datasets clearly presented in a useable and accessible format?

Yes

Are the protocols appropriate and is the work technically sound?

Yes

Reviewer Expertise:

My areas of research are the documentation and description of South American indigenous languages, language typology, and historical linguistics.

I confirm that I have read this submission and believe that I have an appropriate level of expertise to confirm that it is of an acceptable scientific standard.

# Response

**Published:** 2022-09-20

**Authors:** Johann-Mattis List

**Affiliations:** University of Passau, Germany

Thanks a lot for this very thoughtful review. We will respond to the critical points in a detailed reply, once we have received additional reviews for this study. We hope that we will be able to address problems mentioned by the reviewer in a revised version or at least to make the problematic points mentioned more transparent in our data description and avoid them in future work or design plans to make up for them in the future.

# Response

**Published:** 2022-12-02

**Authors:** Johann-Mattis List

**Affiliations:** University of Passau, Germany

We thank the reviewer for this very interesting and encouraging review. We deeply appreciate the time it took to check both paper and data. We agree with the reviewer that the mixing of multiple sources for the same language varieties constitutes a problem of the current database. While we cannot change this problem at the moment, we will try to avoid it in the future. We have added a short paragraph at the end of our study, where we emphasize this problem more transparently and recommend colleagues who plan similar data collections in the future, to make sure to avoid these problems.

 We also agree with the reviewer that we missed a chance by not listing the original transcription and our modification in all cases. Again, we have added a short paragraph to the discussion of our study to make sure readers can take this as a recommendation to try and avoid these problems from the beginning when carrying out similar projects of data collection.
